# The Surgical Diseases of the Genito-Urinary Tract, Venereal and Sexual Diseases

**Published:** 1900-03

**Authors:** 


THE SURGICAL DISEASES OF THE GENITO-URINARY TRACT, VENEREAL AND SEXUAL DISEASES. A Text-book for Students and Practitioners. By G. Frank Lydston, M. D., Professor of the Surgical Diseases of the Genito-Urinary Organs and Syphiology in the Medical Department of the State University of Illinois; Professor of Criminal Anthropology in the Kent College of Law; Surgeon-in-Chief of the Genito-Urinary Department of the West Side Dispensary. Fellow of the Chicago Academy of Medicine; Fellow of the American Academy of Political and Social Science; Delegate from the United States to the International Congress for the Prevention of Syphilis and the Venereal Diseases, held at Brussels, Belgium, September 5, 1899, etc. Illustrated with 233 Engravings. 6½x9¾ inches. Pages xvi-1024. Extra Cloth, $5.00, net. Sheep or Half-russia, $5.75 net. The F. A. Davis Co., Publishers, 1914-16 Cherry St., Philadelphia.

The author has given in this book a practical survey of the field of genito-urinary and venereal diseases. He has followed very closely his lectures as delivered in the Medical Department of the University of Illinois. The work is intended more particularly for students and general practitioners. The style is Lydston's, and that is saying a great deal. Every sentence is easy and comprehensive, and with every subject the reader is impressed that the author wrote from experience rather than theory. It is one of the best books of the year, and should become very popular with all classes of practitioners.

